# Testicular mixed germ cell tumor presenting with seizure as the initial symptom: a case report and literature review

**DOI:** 10.1590/S1677-5538.IBJU.2018.0523

**Published:** 2019-07-27

**Authors:** Syuan-Hao Syu, Chia-Lun Chang, Hung-Jen Shih

**Affiliations:** 1 Department of Urology, Wan Fang Hospital, Taipei Medical University, Taipei City, Taiwan; 2 Department of Urology, School of Medicine, College of Medicine, Taipei Medical University, Taipei City, Taiwan; 3 Department of Hematology, Wan Fang Hospital, Taipei Medical University, Taipei City, Taiwan

**Keywords:** Testis, Testicular Germ Cell Tumor 1 [Supplementary Concept], Neoplasm Metastasis

## Abstract

Most patients with testicular germ cell tumor present with a painless scrotal mass. We report a 19-year-old patient who presented with neurological complains. Rapid clinical progression to coma was noted during the staging work up. A diagnosis of testicular mixed germ cell tumor with multiorgan metastasis (lymph node, lung, liver and brain) was made. Patients with brain metastasis should receive chemotherapy alone or combined with surgery or radiotherapy. Because the clinical symptoms deteriorated quickly, surgery was used upfront followed by chemotherapy and radiotherapy for the brain tumor. After the first stage of treatment, the clinical symptoms, tumor markers and imaging findings were improved. The residual brain tumor was eliminated by chemotherapy, and only sparse degenerated tumor cells were noted in the brain tissue. Longer follow up is required to assess the impact of our treatment strategy.

## INTRODUCTION

The worldwide incidence of testis tumors is low (0.5-7.8 per 100,000 men) (1); however, testicular cancer is the most common solid tumor in men 15 to 34 years of age (2). Other symptoms depend on the burden and location of metastatic lesions. Most patients are diagnosed with clinically localized disease at an early stage, which has a high cure rate (3). Testicular germ cell tumors (GCT) are chemosensitive tumors, and there can be excellent survival with widespread metastases (4). Testicular tumor with brain metastasis is rare and portends a poor prognosis (5). The optimal treatment for testicular GCT with brain metastasis is unknown due to the rarity of this scenario. We report a case of widely metastatic testicular GCT in a patient who presented with neurologic symptoms and rapid clinical progression to coma.

## CASE PRESENTATION

**Figure 1:**
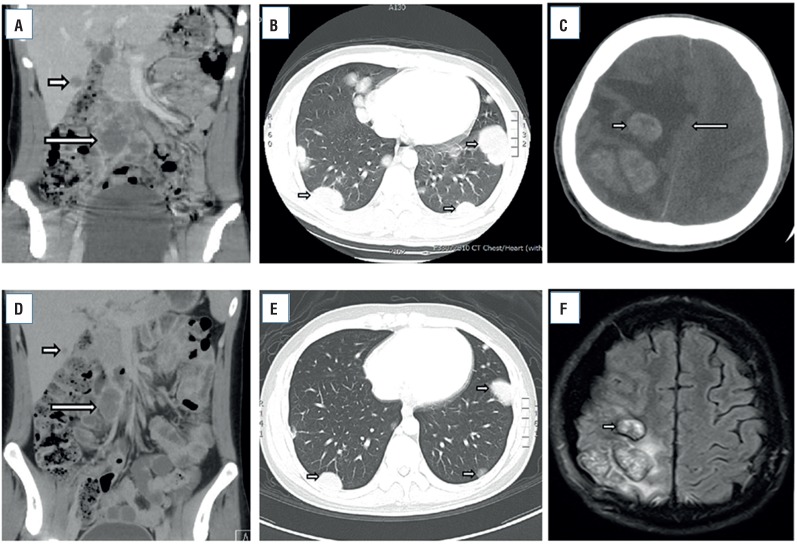
A) abdominal and pelvic CT showed liver (short arrow) and lymph node metastasis (long arrow) before chemotherapy treatment; B) chest CT showed multiple lung metastatic tu-mors before chemotherapy treatment (short arrows); C) Brain computed tomography (CT) sho-wed three heterogeneous, enhanced brain tumors with midline shift (short arrow: brain tumor, long arrow: midline shift); D) abdominal and pelvic CT showed shrinkage of the liver (short ar-row) and lymph node metastases (long arrow) after four courses of chemotherapy treatment; E) chest CT showed that multiple lung metastatic tumors had decreased in size after four courses of chemotherapy treatment (short arrows); F) brain MRI showed shrinkage (short arrow) and enhanced homogeneity of the brain tumor components without midline shift after completion of four courses of chemotherapy treatment.

A 19-year-old male presented with a single seizure attack. Brain computed tomography (CT) revealed three brain tumors that were suspected metastases. The largest tumor measured 2.3 cm. According to the patient's statement, a right testicular mass was noted 6 months prior. A hard testicular mass was palpable in the right testis, and scrotal ultrasonography showed a heterogeneous mass of 5.4×3.7 cm. The tumor markers for testicular tumor were all elevated (LDH, 1483 U/l; AFP, 810.88 ng/ml; and b-HCG, 506166 mIU/ml). Abdominal and pelvic CT revealed multiple bilateral pulmonary metastatic tumors, retroperitoneal lymph node enlargement (para-aortic, aortocaval and iliac chain lymphadenopathy; the largest lymph node measured 4.72×4.00 cm) and a metastatic liver tumor (S4: 2 cm, S6: 2.5 cm) (Figures 1A and B). Because no further seizure attack occurred after treatment via medication, we immediately performed a right radical orchiectomy and port-a-cath insertion (for chemotherapy treatment) under spinal anesthesia on the first day of hospitalization. The testicular tumor pathology showed a mixed GCT (embryonal carcinoma: 40%, post-pubertal type yolk sac tumor: 32%, choriocarcinoma: 25%, and teratoma: 3%). However, left upper and lower extremity weakness was noted on the third postoperative day, and brain CT showed progression of brain edema with midline shift (Figure-1C). The Eastern Cooperative Oncology Group (ECOG) score was 4. Due to rapid clinical deterioration, surgical intervention was performed to decrease intracranial pressure. A craniectomy with brain tumor biopsy was performed, and the brain biopsy confirmed a non-seminomatous germ cell tumor (NSGCT).

**Figure 2:**
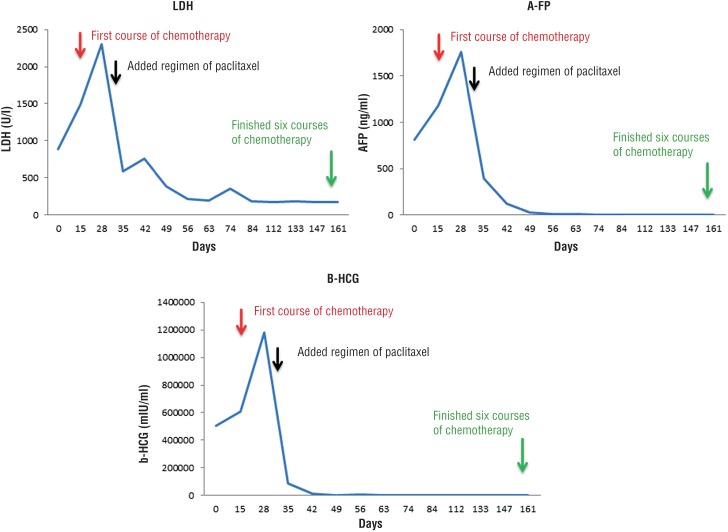
The tumor marker changes from the initial diagnosis to the end of six courses of chemotherapy.

Systemic chemotherapy (etoposide and cisplatin) was administered, and radiotherapy was performed for the brain tumor and whole brain (whole brain 30 Gy and tumor area 10 Gy). However, the tumor markers increased (LDH: 2301 U/l, AFP: 1757.7 ng/ml, b-HCG: 1177684 mIU/ml), and a change in consciousness (Glasgow Coma Scale: E1V1M1) was noted. Brain CT showed an increase in tumor size (3.2 cm) with compression of the brain stem. An emergency craniectomy was performed. The chemotherapy regimen was changed by adding paclitaxel due to its greater ability to penetrate the blood-brain barrier (BBB). After intensive care, the patient's visual condition and left paraplegia, due to compression by the brain tumor, improved. After a total of six courses of chemotherapy and radiotherapy, follow-up CT and magnetic resonance imaging (MRI) showed residual tumor but a decrease in size of the lung, liver, retroperitoneal lymph node and brain tumors (Figure 1D-F) as well as a decrease in tumor markers (LDH: 174 U/l, AFP: 2.30 ng/ml, and b-HCG: 473.80 mIU/ml; Figure-2). A decrease in ECOG score (from 4 to 1) was also noted. The residual brain tumor was removed without complication at one month after completion of chemotherapy and radiotherapy, and the pathology results showed only scant degenerated tumor cells in the brain tissue. Because of the residual organ metastasis and persistent elevation of b-HCG, second-line chemotherapy with vinblastine, ifosfamide and cisplatin was continuously administered.

## DISCUSSION

Most testicular cancers are GCTs, which are classified as seminoma and NSGCTs. With NSGCTs being most common (64.7%) (6). Compared to testicular seminoma, NSGCT has a poor prognosis (7). Embryonal and choriocarcinoma are two types of NSGCTs with a poor prognosis due to their tendency for early hematogenous and lymphatic spread (8). Our patient had four types of NSGCTs in his testis (embryonal carcinoma, post-pubertal type yolk sac tumor, choriocarcinoma, and teratoma). The rapid clinical progression of our patient might be related to the aggressive GCT components of the testicular tumor, large tumor burden and multiple metastatic locations.

Distant metastasis is rare in testicular GCTs, especially brain metastasis (1~2% of testicular GCTs) (5). For distant metastasis, chemotherapy is typically used as an adjuvant treatment after radical orchiectomy. (9). However, chemotherapy for metastatic brain tumors has a poor penetration rate due to the BBB (5). Therefore, radiotherapy or surgical intervention can be used as adjunctive therapy to chemotherapy. For advanced testicular cancer patients, the sequence of treatment modality is controversial. Salvati et al. reported that surgical intervention could initially be recommended if a metastatic brain tumor can possibly be resected (10). In our case, systemic chemotherapy following initial radical orchiectomy and port-a-cath insertion was planned. However, a rapid neurologic decline precipitated a change in the treatment plan involving brain surgery and radiotherapy.

According to the National Comprehensive Cancer Network guideline (11), chemotherapy with bleomycin, etoposide and cisplatin (BEP) is suggested as the first-line treatment for advanced NSGCT. For the initial treatment of brain metastasis of testicular cancer, no significant difference in the efficacy of BEP compared to that of vinblastine, ifosfamide, and cisplatin has been reported (12). In addition, we were concerned about the side effects of bleomycin, such as pulmonary fibrosis, in our patient, who had multiple metastatic lung tumors. In our case, we chose EP combined with radiotherapy for the initial treatment of the brain tumor, but paclitaxel was added because the clinical situation had changed due to the progressive elevation of tumor markers. We selected paclitaxel because of its greater ability to penetrate the BBB, and its efficacy as a regimen for GCTs (13). In our case, decreased tumor markers and follow-up imaging studies indicated decreases in the liver, lung and retroperitoneal lymph node metastatic lesions. The size of the brain tumors remained stable, but the edema improved. The clinical symptoms dramatically improved (the ECOG score from 4 to 1). After brain tumor resection was performed one month later, only scant degenerated tumor cells in the residual brain tumor area were noted, further demonstrating the good response of the brain tumors to our treatment protocol. Because of the residual tumors in the lung, liver and retroperitoneal lymph nodes and because the b-HCG level did not return to a normal range, second-line chemotherapy with vinblastine, ifosfamide and cisplatin was used for continuous treatment (14).

## CONCLUSIONS

The patient was diagnosed with widely metastatic testicular GCT after initially presenting with neurologic manifestations. The disease was controlled by multiple treatment modalities (chemotherapy, radiotherapy and brain surgery).
